# Enhancement of Transport Selectivity through Nano-Channels by Non-Specific Competition

**DOI:** 10.1371/journal.pcbi.1000804

**Published:** 2010-06-10

**Authors:** Anton Zilman, Stefano Di Talia, Tijana Jovanovic-Talisman, Brian T. Chait, Michael P. Rout, Marcelo O. Magnasco

**Affiliations:** 1 Theoretical Biology and Biophysics Group and Center for Nonlinear Studies, Theoretical Division, Los Alamos National Laboratory, Los Alamos, New Mexico, United States of America; 2 Laboratory of Yeast Molecular Genetics, The Rockefeller University, New York, New York, United States of America; 3 Laboratory of Mass Spectrometry and Gaseous Ion Chemistry, The Rockefeller University, New York, New York, United States of America; 4 Laboratory of Cellular and Structural Biology, The Rockefeller University, New York, New York, United States of America; 5 Laboratory of Mathematical Physics, The Rockefeller University, New York, New York, United States of America; Max-Planck-Institut für Informatik, Germany

## Abstract

The functioning of living cells requires efficient and selective transport of materials into and out of the cell, and between different cellular compartments. Much of this transport occurs through nano-scale channels that do not require large scale molecular re-arrangements (such as transition from a ‘closed’ to an ‘open’ state) and do not require a direct input of metabolic energy during transport. Nevertheless, these ‘always open’ channels are highly selective and pass only their cognate molecules, while efficiently excluding all others; indeed, these channels can efficiently transport specific molecules even in the presence of a vast excess of non-specific molecules. Such biological transporters have inspired the creation of artificial nano-channels. These channels can be used as nano-molecular sorters, and can also serve as testbeds for examining modes of biological transport. In this paper, we propose a simple kinetic mechanism that explains how the selectivity of such ‘always open’ channels can be based on the exclusion of non-specific molecules by specific ones, due to the competition for limited space inside the channel. The predictions of the theory account for the behavior of the nuclear pore complex and of artificial nanopores that mimic its function. This theory provides the basis for future work aimed at understanding the selectivity of various biological transport phenomena.

## Author Summary

Various channels and transporters shuttle molecules into and out of the cell, as well as between different cell compartments. Such channels have be selective, i.e. to pass only certain molecular species in a given direction, while efficiently blocking the passage of all others. Transport properties of some channels (e.g. ion channels), have been extensively studied. However, the mechanisms of channels that conduct larger molecules, such as the nuclear pore complex, which gates all transport between the cell nucleus and the cytoplasm, are less understood. In particular, it is still not clear how such channels can efficiently transport their specific molecules even in the presence of a vast excess of non-specific molecules that potentially could clog the channel. Understanding how such channels work is also important for technological applications, such as design of artificial nano-filters. In this paper, we propose a mechanism of selectivity of such channels in the presence of vast amounts of background molecular noise. The predictions of the theory account for the behavior of the nuclear pore complex and of artificial nanochannels that mimic its function. The theory provides the basis for future work aimed at understanding the selectivity of transport through various biological and artificial channels.

## Introduction

Living cells require the efficient and selective trafficking of molecules through various transport channels [1], [2]. Some transporters require large conformational changes, involving transitions from ‘closed’ to ‘open’ states and a direct input of metabolic energy during transport [1]. However, many other transporters provide efficient and selective transport without large conformational changes and without a direct input of metabolic energy during transport. Examples of the latter transport mechanisms include selective permeability of porins [3]–[8], transport through the nuclear pore complex (NPC) [9]–[13], and the access of ligands to the active sites of certain enzymes [14]. In the context of transport through the NPC, such a mode of transport has been termed ‘virtual gating’ [12], [15]. Ion channels also belong to this class of transporters, although factors specific to ion channels set them beyond the scope of the present work [16]. Recently, artificial molecular nano-channel devices have been built that mimic and utilize the principles upon which the function of natural transporters is based [17]–[24]. In this paper, we focus on an artificial nano-molecular channel that mimics the functioning of the NPC [23], as the mimic provides important insights into the function of the underlying biological channel.

Despite their variety, such natural and artificial transporters appear to share common mechanisms of transport selectivity and efficiency. They commonly include a channel or a passageway, through which molecules translocate by diffusion [2]–[24]. Often, selective transport involves transient interactions of the transported molecules with corresponding receptors inside the channel [2]–[24], which leads to transient trapping of the transported molecules in the channel. The selectivity mechanisms of such channels are still a matter of debate. A crucial insight is that the channel geometry, even in the absence of any physical barrier for particle entrance, the probability of a particle to transolcate through a channel is low [25], [26]. Transient trapping increases the probability of transport of individual molecules and thus enhances the transport. Related effects arise in selective membrane transport, known as ‘facilitated diffusion’ in that context [2], [15], [25]–[32]. However, if molecules spend too much time in the channel, the rate at which they leave the channel is lower than the rate at which they attempt to enter - which leads to jamming and a decrease of transport. Hence, transport efficiency can be optimized by tuning the interaction strength of the transported molecules with the channel. The selectivity of such channels can thus be based on the differences in the trapping times of the optimally interacting molecules compared to others [5], [15], [31], [33]–[37]. It is important to emphasize that the efficiency and selectivity of transport are determined not by the equilibrium interaction strength of the molecules with the channel *per se*, but by the *rates* at which the molecules enter, translocate through, and exit from the transport channel [15], [34], [36], [37]. These rates are in many cases determined by the strength of the interactions with the transport device, but can be also determined by its geometry [2]–[23]. For instance, the trapping times inside a channel can be limited by diffusion through convoluted passages inside the channel (e.g. in zeolites), known as ‘entropic trapping’ [38]–[41]. Theories based on these ideas provide an adequate explanation of transport selectivity of artificial nano-channels for single species transport (for instance, [42]).

However, in nature (and in order to be useful in many technological applications such as molecular sorters) the selected molecules have to be transported through a channel in a vast background of other molecules, many of which can interact weakly and non-specifically with the transport channel. Thus, transport channels have to be able to constantly select their cognate molecules from such a background. It is still not clear precisely how biological and artificial channels can perform selective transport under such conditions, but any useful theoretical description must take into account this non- specific competition. It is likely that various mechanisms can contribute to selectivity. For instance, in some cases, the selectivity arises from the presense of a physical or energetic barrier for the entrance of non-specific molecules into the channel [27]–[29].

In this paper we focus on the universal selectivity properties of channels, which do not depend on the specific molecular details pertinent to each specific transporter. We show that highly selective transport is possible in the presence of non-specific competition even when the non-specific molecules are free to interact with and enter into the channel. We study the case of a mixture of two molecular species of different trapping strenghts attempting to traverse the channel. Our model relies on only two essential ingredients: transient trapping of the molecules in the channel and inter-molecular competition for the limited space inside the channel. Analysis of the model reveals a novel kinetic mechanism of the enhancement of transport selectivity through narrow channels, which relies on the sequential exclusion of weakly trapped (low affinity) non-specific molecules from the channel due to competition with strongly trapped (high affinity) cognate molecules that spend a longer time in the channel. Comparison of the theoretical predictions with experimental data shows that the predicted mechanism accounts for the transport selectivity observed in an artificial nano-channel that mimics the NPC. Due to its generality, the proposed mechanism of selectivity is expected to play a role in various biological and artificial nano-channels.

## Results

We model transport through a narrow channel in the framework of a general kinetic theory [5], [15], [36], [38], [42]–[47]. The channel is modeled as a sequence of positions (‘sites’). The movement of particles (molecules) through the channel is described as diffusive hopping from one position to the next, subject to the condition that each position can accommodate only a finite number of particles – i.e., a particle cannot hop if a neighboring position is fully occupied. This latter assumption models the limited space inside the channel [15], [36], [38], [42], [48]. Such a simplified treatment captures the essentials of hindered diffusion through narrow channels, and indeed has been successfully used for the explanation of transport properties of various channels [15], [25], [26], [31], [33]–[36], [38], [41], [42], [49]–[52].

### The ‘one site’ channel case

**Figure 1:**
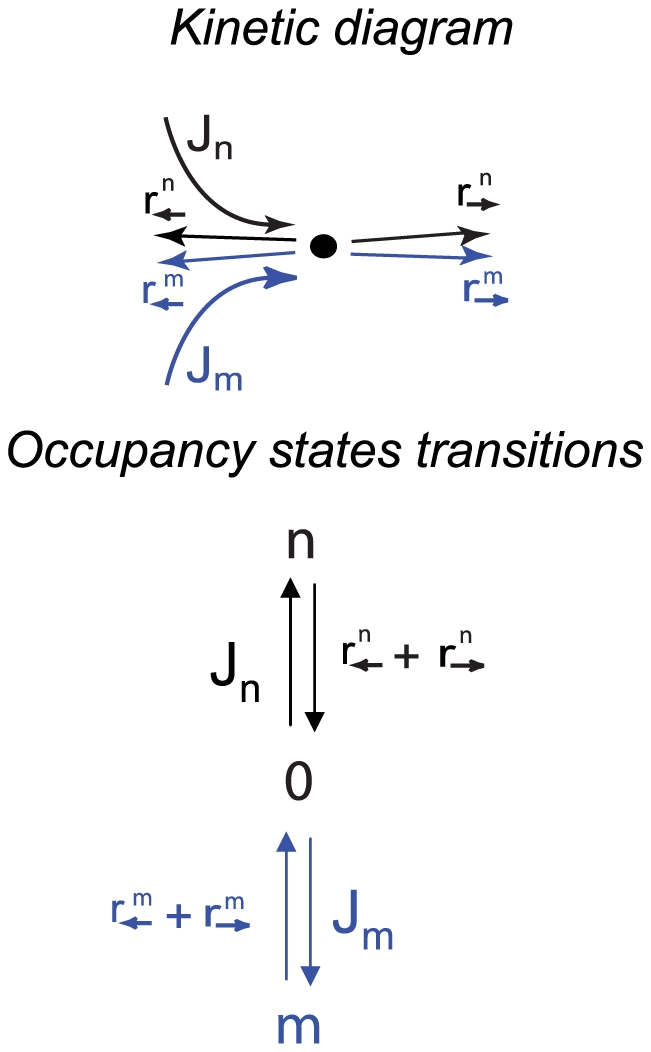
Kinetic scheme of a ‘one-site’ channel. **Top.** Two species of particles, *m* and *n*, enter the channel with fluxes 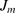 and 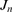, if the channel is not occupied. Upon entry, they can either hop forward with rates 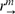 or 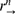 respectively, or hop backwards with rates 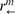 and 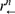, respectively. **Bottom.** Alternative occupancy representation of the transport kinetics as transitions between the three possible occupancy states: occupied by an 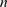 -type particle, or occupied by an 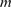-type particle, or unoccupied.

Let us first consider a ‘one-site’ channel model (Fig. 1). All the details of the potentially complicated kinetics of transport through the channel are absorbed into the forward and backward exit rates 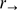 and 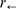. These exit rates can be thought of as ‘off’ rates for the release of the particles from the channel. Particles of two different species (denoted as *n* and *m*) attempt to enter the channel from the left (Fig. 1). Particles of species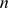 enter the channel with the rate 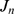 if the channel is unoccupied, exit at the right end with the rate 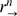, or return to the left side with the rate 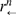. The respective rates for the other species, particles of type 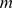, are 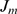, 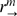 and 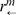 (Fig. 1). The channel can be in three states: occupied by an 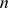-species particle, occupied by an 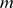-species particle, or un-occupied, with the respective probabilities 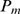, 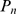, and 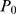. This scheme explicitly allows only one particle of any type to be present in the channel at any time. In other words, if the channel is occupied by a particle of either species, other particles cannot enter until the residing particle hops out. Note the parallel between transport through such one-site channel and the Michaelis-Menten kinetics of enzymatic reactions – the channel is analogous to the enzyme molecule, while the transported particles are analogous to the substrates. The master equation describing the kinetics of transport through the channel is [2], [43] :
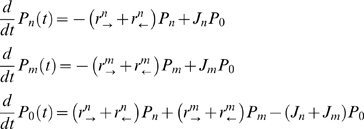
(1)Note that 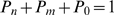, (so that 
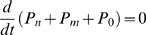
) because the channel has to be in some state. Transmitted fluxes to the right of the particles of each type are 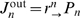 and 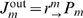, respectively. Solving equations (1), we get:
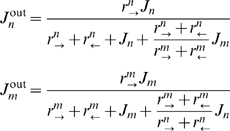
(2)


We define the *efficiency* of transport as the ratio of the transmitted flux to the impinging flux, 
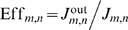
. However, not all the particles that attempt to enter the channel succeed, because the channel is occupied with the probability 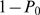. The transport efficiency is thus different from the translocation *probability* of a particle that has entered the channel to exit on the right - a fact that will become important below. Mathematically, the translocation probability is defined as 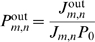.

From eq. (2), in the absence of competition, when particles of only one type are present (say 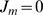), in the limit of small currents (when 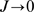), the efficiency and the probability are identical and equal to 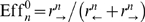. In the case when both particle species are competing for space in the channel, from equation (2), the ratio of transport efficiencies of *m*-species and *n*-species is
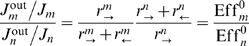
(3)


Thus, the transport efficiency of the particles of each type through a single-site channel is not influenced by the presence of particles of the other type. As we show below this is not so for channels that can accommodate more than one particle.

### Long channels: the ‘N-site’ channel case

Selectivity conditions change when one considers transport in a mixture of two different species of molecules in longer channels, where the molecules can interfere with each other's passage through the channel. The main result is that in the presence of more strongly trapped species, the transport of more weakly trapped species is strongly inhibited, compared to the case when they are present alone.

#### Setting up the model

**Figure 2:**
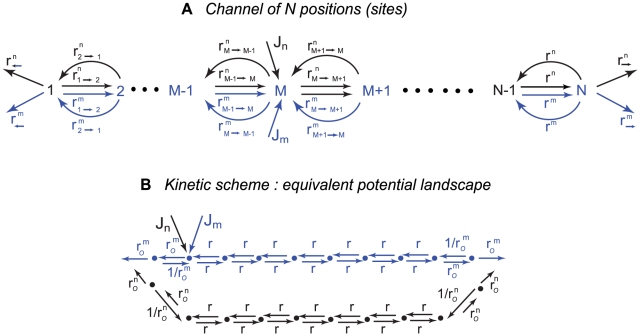
Kinetic scheme of transport through an N-site channel. **A.** The channel is represented as a chain of 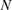 positions. The blue arrows denote the transition rates of the particles of species 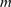, which enter the channel at a position 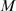 with an average rate 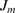, if its occupancy is smaller than the maximal allowed. The black arrows denote the transition rates of particles of species 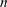 that also enter at site 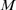 with an average rate 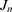. **B.** The kinetic profile example used for the simulations presented in Fig. 4. One species (*m*) of particles – shown in blue - interacts weakly with the channel, and is trapped inside only weakly. The otherspecies of particles (*n*) – shown in black – is strongly (but transiently) trapped in the channel, as modeled by lower exit rate 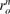 and higher ingress rate 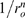 near the channel entrance at position 2.

Analogous to a single-site channel, a longer channel that may contain several particles simultaneously can be represented by a sequence of 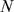 positions (sites): 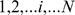. The ratio of channel diameter to particle size is modeled by allowing up to a maximal number of particles 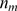 to occupy a given position. Particles of both species are stochastically deposited at a position *M* (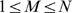), with average fluxes 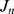 and 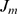 respectively, and enter the channel if the occupancy of the entrance site is less than the maximal 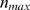. Once inside the channel, a particle of species *n* present at an internal position 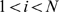 can hop to either one of the neighboring positions 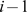 and 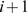, at an average rate 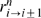, if the either site is not fully occupied. From the exit positions 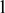 or 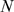 the particle can hop to leave the channel, at an average rate 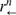 or 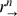 respectively, or hop to the position 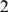 (or 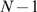 respectively), if the latter is not fully occupied, with an average rate 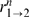 (or 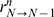, respectively). Similarly, particles of species 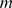 can hop between adjacent positions with the rate 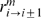 and exit the channel with rates 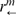 on the left and 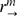 on the right. A general kinetic scheme of such transport is shown in Fig. 2. We emphasize that the ‘sites’ do not necessarily correspond to actual physical binding sites, but are merely a convenient computational tool to describe hindered diffusion [15], [36]–[38], [42], [44], [48], [51], [53]. As mentioned above, the rates of hopping through and exit from the channel are influenced by many factors, including the binding affinity of the particles in the channel and the channel geometry. In the case when the rates are determined only by the binding energies of the particles inside the channel, they are given by the Boltzmann-Arrhenius expression 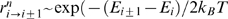 where 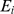 is the energy of a particle at site *i* [43]. In principle, an analytical solution for a long channel can be obtained using the same method as described above for the ‘one -site’ channel; such an analytical solution for a channel containing only two sites is shown in the Supporting Information (Sec. 1 in Text S1, and Figs. S1 and S2). However, a channel longer than two sites is easier to treat using computer simulations. Therefore, we have simulated the hopping process described above using a variant of the Gillespie-Bortz-Kalos-Leibowitz (Kinetic Monte Carlo) algorithm [15],[42],[54]–[56]. Detailed description of the algorithm and the actual code are given in the Supporting Information (Sec. 4 in Text S1).

Here, we show the results for the kinetic landscape shown in Fig. 2 **B**. The 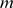-species (blue) is weakly (or not at all) trapped in the channel. The 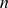-species (black) is strongly trapped in the channel - i.e., their exit (‘off’) rate from the channel is lower than that of the *m*-type particles, 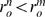. In this example, the impinging fluxes of both species enter at site 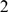 and leave at either site *1* or *N*, which models a case when the exit site does not necessarily co-localize with the entrance position (as may be found in some biological or artificial channels) [2]–[11] or diffusion of the particles outside the channel; see Supplementary Information for more examples. In the simulations, we keep the exit rate of the strongly trapped species 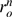 fixed, and vary the exit rate of the weakly trapped species 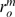.

#### Single species: the role of trapping

**Figure 3:**
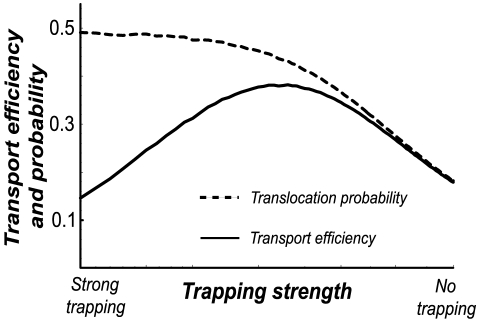
Transport efficiencies and probabilities for a single species. Transport efficiency (black line) and translocation probability (dotted line) for single species (say, *n*-species in the absence of m-species) as a function of the trapping strength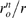, for *J/r = 0.01*. The transient trapping increases the probability that the particles translocate through the channel after they have entered (dotted line). This leads to an accompanying increase in transport efficiency; however for trapping that is too strong, particles residing in the channel prevent the entrance of new ones and transport efficiency decreases.

We first review the selectivity conditions when only one species is present (say only *n*-species, so that 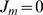) [15], [25], [26], [31], [33], [36], [38], [42], [49], [51], [57]. The inter-particle competition for confined space inside the channel affects both their ability to enter the channel and translocate through it. Therefore, as above, one has to distingush between two characteristics of transport: the transport efficiency, and the translocation probability. The former is the fraction of the impinging current *J* that traverses the channel. The latter is the fraction of those particles that have actually entered the channel on the left that reach the other end. The results are summarized in Fig. 3, which shows the efficiency and the probability of transport as a function of the trapping strength. It shows that the probability of transport initially increases with the trapping strength, even when the particles interfere with each other's passage. However, at high trapping strengths, the particles spend too much time in the channel, so that the entrance becomes blocked. This prevents the entrance of new particles and leads to a decrease in the transport efficiency – the channel becomes jammed. This provides a natural definition of a jamming transition as a point where the transport efficiency starts to decrease (see Fig. 3). Overall, for exit rates above the jamming transition, the more weakly trapped (non-specific) particles are transported less efficiently than the more strongly trapped (specific) ones, but still their flux is not negligible [15], [25], [26], [31], [33]–[35], [42], [49], [50]. However, as we will see below, the difference in the transport of the weakly and strongly trapped particles is enhanced much more when they are present in a mixture.

#### Selectivity is enhanced by inter-species competition

**Figure 4:**
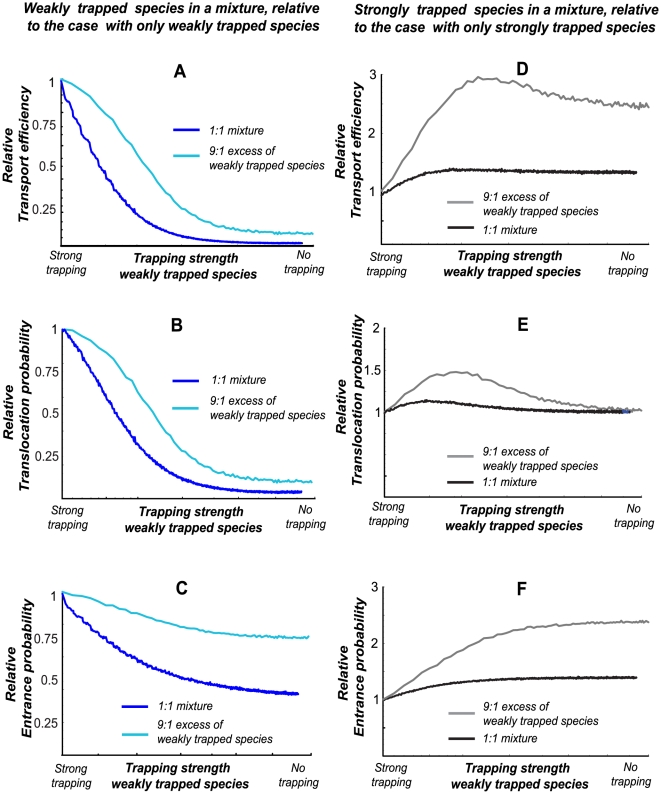
Selectivity enhancement in a mixture of two species. The left panels describe the transport of a weakly trapped species in a titrated mixture with the strongly trapped species, relative to the case when only a weakly trapped species is present. The right panels describe the transport of a strongly trapped species in the same mixture relative to the case when only a strongly trapped species is present. In all panels the total combined flux of the particles is 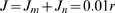; log-linear scale in all panels. Transport of weakly trapped particles is inhibited by competition with more strongly trapped ones: panels **A, B, C.** (**A**) Efficiency of transport of the weakly trapped species (*m*) in competition with the strongly trapped species (*n*), relative to the case when the weakly trapped species is present alone in the same concentration, 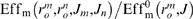 (**B**) Probability of translocation through the channel of a particle of the weakly trapped species, relative to the case when they it is present alone in the same concentration, 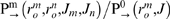. (**C**) Probability to enter the channel of the weakly trapped species, relative to the case when it is present alone in the same concentration, 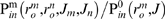. In all panels **A, B, C,** the blue line represents 1∶1 mixture (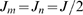) and the turqouise line represents 9∶1 excess of the weakly trapped particles (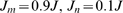). Transport of the strongly trapped species is enhanced by competition with athe weakly trapped species: panels **D, E, F**. (**D**) Efficiency of transport of the strongly trapped species (*n*) in competition with the weakly trapped ospecies (*m*), relative to the case when the strongly trapped species is present alone in the same concentration, 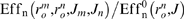. (**E**) Probability of translocation through the channel of the weakly trapped species, relative to the case when it is present alone in the same concentration, 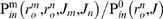. (**F**) Probability to enter the channel of the weakly trapped species, relative to the case when it is present alone in the same concentration, 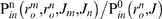. In all panels **D, E, F,** the black line represents 1∶1 mixture (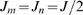) and the gray line represents 9∶1 excess of the weakly trapped particles (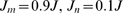).

In the biological context, non-specific molecules interact only weakly with channels whereas specific cognate molecules interact strongly. Predictions of the model for the case when two species directly compete for the space inside the channel, are summarized in Fig. 4. It shows that the transport of the weakly trapped 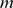-species particles that spend less time in the channel, is greatly inhibited compared to their transport in the single-species case (i.e. in the absense of the more strongly trapped species); see Fig. 4 **A–C**. Even more strikingly, the transport of the strongly trapped *n*-species particles, which spend a longer time in the channel, is *enhanced* by the presence of the weakly trapped competitors: Fig. 4**D**, compared to the case when they are present alone at the same total concentration. (Fig. 4 **D–F**). The difference in the transport efficiencies of the particles of two types increases with the difference in the trapping strength between them. Notably, the inhibition of transport of more weakly-trapped particles, and the enhancement of transport of strongly trapped particles persists even when the incoming flux of the weakly trapped particles is an order of magnitude higher than that of the strongly trapped ones - Fig. 4**A** and **D**.

Why does competition between different particle species enhance the selectivity of the transport? As mentioned above, the overall transport efficiency is influenced by two factors: 1) the ability of a particle to enter the channel in the first place (the entrance site might be temporarily occupied which prevents the entrance of new particles) and 2) the probability of a particle to translocate through the channel, *after* it has entered. As Fig. 4 C and F show, although the entrance to the channel of the more weakly trapped species is somewhat inhibited by the strongly trapped species, compared to the case when it is present alone, this is not the main factor in the overal inhibition of their transport. Rather, the *probability* of the weakly trapped particles *to translocate* through the channel decreases due to competition for space with the more strongly trapped particles (Fig. 4B and E; cf. also Figs. S2 and S3).

**Figure 5:**
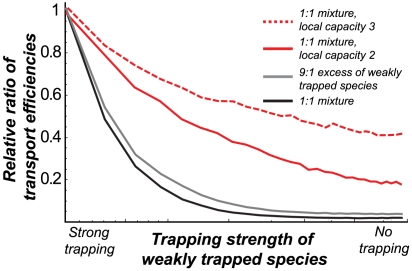
Competition inhibits the transport of the weakly trapped species even in wide channels. Ratio of the transport of the weakly trapped species to that of the strongly trapped species with competition, normalized by the ratio of the single-species efficiencies; black line: equal mixture (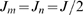) for a channel accommodating up to one particle at each site gray line: 9-fold excess of the weakly trapped species (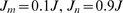) for a channel accommodating up to one particle at each site, 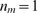 red line: channel accommodating up to two particles at each site (maximal local occupancy 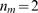), red dotted line : channel can accommodate up to three particles at each site (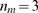). The selectivity enhancement decreases with the channel width; *J = 0.01r*.

These results are summarized in Fig. 5, which shows the ratio of transport efficiencies of the two species. Fig. 5 describes the main result of this paper: when the incoming flux consists of a mixture of particles whose transport times through the channel are different, the selectivity conditions change compared to the single-species case. In the presence of strongly trapped (cognate) particles that spend a longer time inside the channel, the transport of the weakly trapped (non-specific) particles is inhibited relative to the single-species case.

The heuristic explanation for this phenomenon is that the particles that are strongly trapped in the channel block translocation through it. If, during the time when the channel is blocked by a strongly trapped particle present somewhere inside, a weakly trapped particle enters the channel, the latter will with a high probability quickly exit the channel on the left side. If, on the other hand, a strongly trapped particle comes in when the passage to the right side is blocked by another such particle, then, with high probability, it will stay in the pore long enough for the particle that blocks it to pass through.

**Figure 6:**
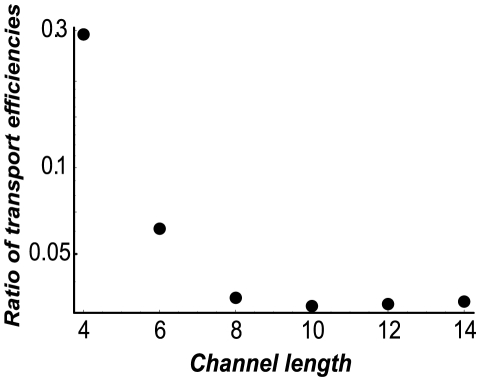
Selectivity enhancement increases with the channel length. Ratio of the transport selectivity of a weakly trapped species to that of a strongly trapped species, as a function of the channel length, 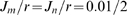.

The inhibition of transport of the more weakly trapped species persists beyond single file transport, when the channel can accommodate several particles at each site as shown in red lines in Fig. 5. However, as the channel width increases, the competition effects become less prominent and the inhibition diminishes. We have also investigated the effect of the channel length on the competition-induced enhancement of selectivity. In accord with the finding that it is the translocation probability through the channel that is mainly affected by the competition, the selectivity enhancement increases with the channel length, as shown in Fig. 6.

**Figure 7:**
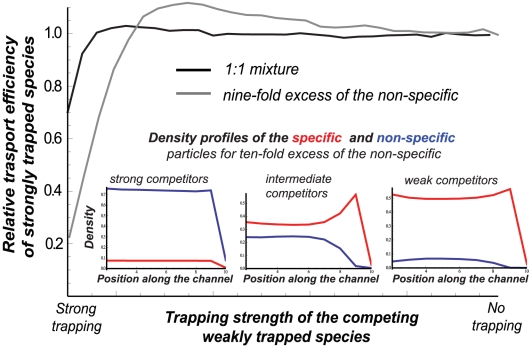
Effect of addition of weakly trapped species on the transport of the strongly trapped species. Relative transport efficiency 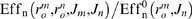 of the strongly trapped species (for 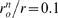
and 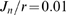) as a function of the trapping strength of the *added* weakly trapped species, when the latter are added in the same concentration 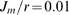 (black) or in tenfold excess 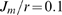 (gray) in the same kinetic profile as in Figs. 3, 4, and 5 (shown in Fig. 2). Addition of the weakly trapped species enhances the transport of the strongly trapped species – see text for discussion. *Inset:* density profile of the specific (red) and non-specific (blue) particles from the channel entrance to the exit for strong (left), intermediate (middle) and (weak) trapping of the non-specific particles present in ten-fold excess.

The effect of the presence of the weakly trapped (non-specific) species on the transport of the more strongly trapped (specific) one can also be examined from a different angle. Namely, instead of *titrating* the strongly trapped species with weakly trapped competitors, so that the total concentration remains constant (as in Fig. 4) one can ask how does the flux of the strongly trapped particles change upon progressive *addition* of the weakly trapped competitors (so that the total combined concentration increases). The result is shown in Fig. 7. Suprisingly, even in this case, the flux of the strongly trapped particles is practically unaffected – or even enhanced – by the presence of non-specific competitors. (See also Sec. 5 in Text S1 and Fig. S5).

The enhancement of transport of the more strongly trapped species by addition of more weakly trapped competitors is somewhat counter-intuitive, as one might expect that increasing the concentration of the non-specific competitors would clog the channel and decrease the flux of the specific particles. In particular, the theory predicts that this enhancement is present only for a certain range of trapping strength of the weakly trapped competitors.

The heuristic explanation of this effect is as follows. When the trapping strength of the non-specific competitors is close to that of the specific molecules, they block the entry and interfere with the entrance of the strongly trapped particles. On the other end, the very weakly trapped (or non-trapped) particles essentially do not penetrate the channel, and the flux of the strongly trapped ones is unaffected by their presence. However, in a certain range of intermediate trapping strengths, the non-specific competitors, although mostly filtered out, still penetrate the channel to a certain degree, accumulating near the entrance (see inset in Fig. 7). This accumulation of the non-specific particles near the entrance hampers the return of the more strongly bound species that are located further down the channnel. This creates an additional effective bias towards the channel exit for the more strongly trapped particles thereby increasing their flux. Thus, the overall effect of the addition of the non-specific particles on the transport of the specific ones is determined by the balance of these two effects: the clogging of the channel entrance and the non-equilibrium variation of the particle density inside the channel.

We note that the inhibition of transport of the weakly trapped non-specific particles by competiton with the specific ones persists even when there are more than two particle species (data not shown). Such non-linear mutual effects of the particles of different species on each other might shed light on opimization of transport by co-transport factors, commonly encountered in biology and also suggest the possibility of creation of artificial ‘nano-valves’ with nonlinear flux rectification properties [58].

We note that the effect described here is a very general mechanism of selectivity of transport through narrow channels and is not limited to a particular fortuitous choice of the kinetic rate constants, being observed for various choices of channel kinetic profiles. Analytical results shown in the Supporting Information support the generality of the mechanism.

### Comparison with experiments

**Figure 8:**
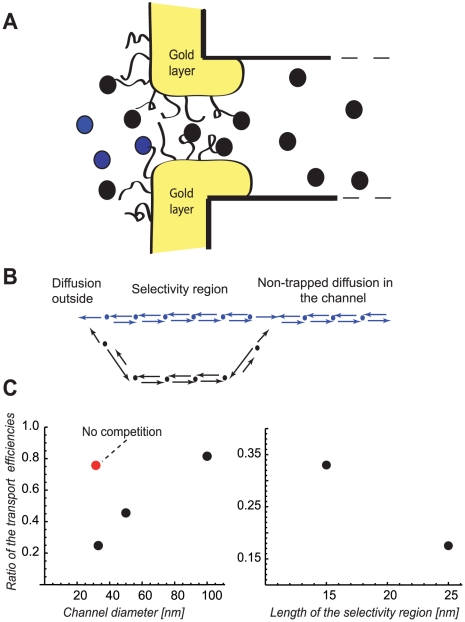
Comparison with experimental data. Panel **A**: schematic illustration of the experimental setup of Ref. [23]. The filamentous proteins (FG -nups) naturally lining the NPC are grafted to the gold layer at the channel opening, thus creating a trapping region, where the specific (NTF2-GST, black circles) and non-specific (BSA, blue circles) molecules compete for space. Approximate diameter of the channel is 33 nm, 50 nm, or 100 nm in different experiments, the Stokes radius of the molecules of both species is ∼3.5 nm. The length of the trapping region is either ∼15 or ∼25 nm. Panel **B**: schematic mapping of the actual channel onto a theoretical model. Panel **C**: Brief summary of the experimental findings of Ref. [23]. This panel shows the ratio of the transport efficiency of the non-binding control protein (BSA) to the transport efficiency of the transport factor NTF2-GST (that binds the FG-nup filaments) for different widths and lengths of the trapping region (normalized by their flux through a non-functionalized channel). In accord with the theoretical predictions, the presence of the specific transport factor inhibits the transport of the non-specific protein and the magnitude of this inhibition decreases with the channel width and increases with the length of the trapping region.

We now turn to comparison of the theoretical predictions with recent experiments on transport through artificial nano-channels that mimic NPC function [23], where many of the parameters discussed above can be varied experimentally. In these experiments, the channels were functionalized with natively unfolded proteins that naturally line the passageway of the NPC (commonly known as the FG-nups). These proteins bind strongly (although transiently) and specifically to nuclear transport factors, but weakly and non-specifically (or not at all) to other proteins (see Fig. 8A). Here we confine ourselves to qualitative comparison with the experiments, to establish the basic mechanisms of selectivity that operate in such channels (Fig. 8B). More quantitative comparisons require more detailed understanding of the local binding-unbinding kinetics of the multiple binding sites on the transport factors to unfolded filamentous proteins within the NPC, as well as realistic modeling of the dynamics of the filaments themselves [59]–[62]. At this stage, the understanding of the mechanistic details of the interactions of the transport factors with the FG-nups and of the movement of the transport factors from one FG-nup to the next is lacking.

Jovanovic-Talisman *et al.* [23] investigated the transport of various nuclear transport factors and of non-specific “control” proteins such as bovine serum albumin (BSA) through the artificial channels described above, and compared the fluxes when they are present either separately or in mixtures. A subset of the experimental results of [23], where the GST tagged nuclear transport factor 2 (NTF2-GST) and BSA were compared, issummarized in Fig. 8**C**. It was observed that the transport of non-binding control protein (BSA) was inhibited by the presence of NTF2-GST, in accord with the theoretical predictions (above). Likewise, the magnitude of the inhibition increased with the length of the trapping region and decreased with the channel width, in accord with the theortical predictions. Thus, the mechanism proposed in this paper account for the experimental results and indicates that selective nano-filters can be built relatively simply, using just the basic stochastic kinetics of the transport process and competition for space inside the channel.

## Discussion

In nature, transport channels have to select for their cognate cargoes over a vast background of other species that might interact with the channel non-specifically. How can they maintain selective transport in such conditions? It is likely that many different mechanisms of selectivity may be operational in such channels [24], [61]–[64]. Here, we have studied a minimal kinetic mechanism of selectivity enhancement, which relies only on the inherent properties of stochastic transport through narrow channels. The model includes only two essential ingredients: transient trapping of the particles inside the channel, and the competition for the limited space inside the channel. The model predicts that weakly trapped (non-specific) species are effectively excluded from transport through the channel by competition with strongly trapped cognate cargoes that spend more time in the channel. In a mixture of two different species - one that is transiently trapped in the channel longer than the other - the transport of the particles of the more weakly trapped species is strongly inhibited compared to the case when they are present alone. Moreover, the theory predicts that transport of the more strongly trapped species is *enhanced* by the presence of the non-specific competitors. These effects are described in Figs. 4 and 7. In the main, inhibition of non-specific competitor transport is not due to prevention of entrance into the channel. Rather, this inhibition is largely due to the diminished probability of translocating through the channel (and so the increased probability of returning to the entrance compartment) *after* the particle has entered the channel. Remarkably, the transport of non-specific particles is inhibited even if their flux greatly exceeds that of the specifically binding particles.

This selectivity enhancement is a purely kinetic, non-equilibrium mechanism. Notably, it does not require input of metabolic energy [1], [65]–[67], but rather stems from the inherent properties of the stochastic transport process. Thus, this effect is expected to hold for various molecular mechanisms of transport through the channels, channel widths, and particle sizes. Even in channels where other effects may be dominant, the effect described here is likely to play a role. It is important to emphasize that for the purposes of the present theory, it is immaterial as to which physical mechanism determines the rate of “hopping” through the channel and the escape rates – i.e., whether they are determined by the binding energies of the particles inside the channel (as in ion channels or porins) [33], [36], [37], [43], geometrical effects such as entropic trapping [38]–[41], or a mixture of the two (e.g. during transport through the nuclear pore complex and artificial nano-channels [8]–[12], [17]–[24]).

Predictions of our theory are in agreement with recent experiments on transport through artificial nano-channels that mimic the nuclear pore complex function [23] - Fig. 8. Thus, both theory and experiment emphasize the need to always consider non-specific competition when studying transport selectivity of both biological and artificial nano-channels. They also highlight the role that the specifc molecules play in the selectivity – they can be viewed as an essential part of the selectivity mechanism. In their absence, it is possible that the channel can be essentially non-selective and can pass various non-specific molecules; it is the presence of the specific molecules that makes the transport selective. The theory also makes verifiable predictions on how the addition of non-specific molecules affects the transport of the specific ones. We expect that future comparison of the theory with experimental data will lead to further refinements of the theory and elucidation of additional selectivity mechanisms, thus allowing the design of more selective artificial nano-channels. Future questions include the mutual influence between the fluxes of particle species in multi-species case, as well as more detailed modeling of the diffusion of the transport factors through the layer of the FG-nups (in the context of the NPC transport) and the analysis of single molecule tracking experiments [57], [68]–[71]. Finally, our theory can be generalized to describe mechanisms of selectivity in arbitrary signal transduction schemes [67], [72]–[74].

## Materials and Methods

The analytical calculations were perfromed by pencil and paper with the help of Mathematica 5.2 package. Simulations were implemented in C language and run on a cluster of opteron processors under UNIX. The simulation code is presented in Text S1.

## Supporting Information

Text S1 Supporting information text and figure captions.
(0.33 MB DOC)

Figure S1Kinetic diagram of transport of particles of two different species through a two-site channel. **A.** Kinetic diagram of a channel consisting of two positions. **B.** Occupancy representation: transition scheme between nine occupancy states: 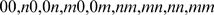.(0.64 MB EPS)

Figure S2Selectivity enhancement in a mixture of two species: two site channel. **A.** Transport of strongly trapped particles is *enhanced* by the competition with the faster ones. The dotted line shows the transport efficiency of the strongly trapped species of exit rate 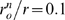 without competition. The blue line shows transport efficiency of the slower particles, in competition with faster particles, as a function of the exit rate of the faster particles 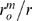, present in the same amount, (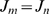). The enhancement occurs even to a higher degree in the 9∶1 excess of the fast particles (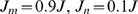) - turquoise line. **B.** Transport of the weakly trapped species is inhibited by the competition with the stringly trapped one. The dotted line shows the transport efficiency of the weakly trapped species as a function of their exit rate without competition. The black line shows transport efficiency of the weakly trapped particles as a function of their exit rate 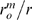, in competition with strongly trapped particles whose exit rate is kept fixed at 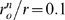, present in the same amount, (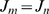). Dotted line- no competition. The inhibition occurs even in the 9∶1 excess of the fast particles (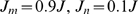) - gray line. **C.** The probability of a particle of weakly trapped species to translocate through the channel is diminished in the presence of the slower particles. By contrast, the probability of a particle of strioingly trapped species to translocate through the channel is enhanced in the mixture. Dotted line - no competition, black line- 1∶1 mixture (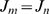), gray line: 9-fold excess of the faster particles (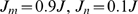). **D.** Ratio of the transport efficiency of the weakly trapped species to that of the strongly trapped species. Dotted line- no competition. Black line∶equal mixture (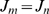), gray line: 9-fold excess of the weakly trapped particles (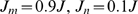). In all panels 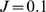.(1.47 MB EPS)

Figure S3Selectivity enhancement in a mixture of two species: long channel. In all panels the exit rate of the strongly trapped species is kept fixed 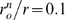 and the total flux 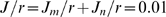 **A.** The blue line shows transport efficiency 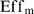 of the weakly trapped particles as a function of their exit rate 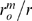, in competition with strongly trapped particles present in the same amount, (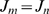). Dotted line- no competition. Turquoise line – 9∶1 excess of the weakly trapped species 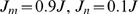. **B.** Probability of translocation through the channel of the weakly trapped species 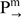. **C.** Probability to enter the channel of the weakly trapped species, 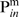. Dotted line- no competition. Turquoise line – 9∶1 excess of the weakly trapped species 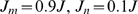. **D.** Efficiency of transport of the strongly trapped species in competition with more weakly trapped one, 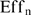. **E.** Probability of translocation through the channel of the strongly trapped species, 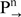 **F.** Probability of the strongly trapped species to enter the channel, 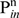. In all panels **D, E, F,** the black line represents 1∶1 mixture (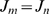) and the gray line represents 9∶1 excess of the weakly trapped particles (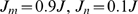). Note that the absolute value of the entrance probability is identical for both species (panels **C** and **F**).(2.68 MB EPS)

Figure S4 Sensitivity to the choice of the kinetic profile. Ratios of the transport efficiency of the weakly trapped species to the transport efficiency of the strongly trapped species for the different kinetic profiles shown in the insets to each panel for different values of the exit rate of the strongly trapped species. See text in Section 3 of Text S1 for discussion.
(2.09 MB EPS)

Figure S5Dependence of the selectivity on the concentration. Panel **A:** Transport efficiency of the strongly trapped species as a function of the flux of the weakly trapped species, in the case of *addition*. 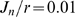, 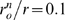, 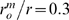 Panel **B:** Ratio of transport efficiencies of the weakly trapped species and the strongly trapped species, relative to the no-competition case, as a function of the total flux of the particles of both species, for the case of *titration*. The ratio of the concentrations is 1∶1, 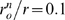, 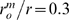, 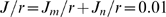.(1.09 MB EPS)

## References

[1] Alberts Mea (1994). Molecular Biology of the Cell: Garland Publishing..

[2] Stein W (1990). Channels, Carriers, and Pumps: An Introduction to Membrane Transport..

[3] Hohmann S, Nielsen S, Agre P (2001).

[4] de Groot BL, Grubmuller H (2001). Water Permeation Across Biological Membranes: Mechanism and Dynamics of Aquaporin-1 and GlpF.. Science.

[5] Lu D, Schulten K, Grayson P (2004). Glycerol Conductance and Physical Asymmetry of the Escherichia coli Glycerol Facilitator GlpF.. Biophys J.

[6] Bezrukov SM, Kullman L, Winterhalter M (2000). Probing sugar translocation through maltoporin at the single channel level.. FEBS Lett.

[7] Nestorovich EM, Danelon C, Winterhalter M, Bezrukov SM (2002). Designed to penetrate: Time-resolved interaction of single antibiotic molecules with bacterial pores.. Proc Natl Acad Sci.

[8] Borgnia MJ, Agre P (2001). Reconstitution and functionalcomparison of purified GlpF and AqpZ, the glycerol and water channels from Escherichia coli.. Proc Natl Acad Sci USA.

[9] Macara I (2001). Transport into and out of the nucleus.. Microbiol Mol Biol Revs.

[10] Stewart M, Baker R, Bayliss R, Clayton L, Grant R (2001). Molecular mechanism of translocation through nuclear pore complexes during nuclear protein import.. FEBS Lett.

[11] Tran EJ, Wente SR (2007). Dynamic Nuclear Pore Complexes: Life on the Edge.. Cell.

[12] Rout M, Aitchison J, Magnasco M, Chait B (2003). Virtual gating and nuclear transport: the hole picture.. Trends in Cell Biology.

[13] Lim R, Aebi U, Fahrenkrog B (2008). Towards reconciling structure and function in the nuclear pore complex.. Histochemistry and Cell Biology.

[14] Luedemann SK, re Lounnas V, Wade RC (2000). How do Substrates Enter and Products Exit the Buried Active Site of Cytochrome P450cam? 1. Random Expulsion Molecular Dynamics Investigation of Ligand Access Channels and Mechanisms.. J Mol Biol.

[15] Zilman A, Di Talia S, Chait B, Rout M, Magnasco M (2007). Efficiency, Selectivity, and Robustness of Nucleocytoplasmic Transport.. PLoS Comput Biol.

[16] Miloshevsky GV, Jordan PC (2004). Permeation in ion channels: the interplay of structure and theory.. Trends Neurosci.

[17] Caspi Y, Zbaida D, Cohen H, Elbaum M (2008). Synthetic Mimic of Selective Transport Through the Nuclear Pore Complex.. Nano Lett.

[18] Lee SB, e al (2002). Antibody-based bio-nanotube membranes for enantiomeric drug separations.. Science.

[19] Jirage KB, Hulteen JC, Martin CR (1997). Nanotubule-Based Molecular-Filtration Membranes.. Science.

[20] Kohli P (2004). DNA-Functionalized Nanotube Membranes with Single-Base Mismatch Selectivity.. Science.

[21] Iqbal S, Akin D, Bashir R (2007). Solid-state nanopore channels with DNA selectivity.. Nature Nanotech.

[22] Savariar E, Krishnamoorthy K, Thayumanavan S, Nanotechnology N, Australia M (2008). Molecular discrimination inside polymer nanotubules.. Nature Nanotech.

[23] Jovanovic Talisman T (2009). Artificial nanpores that mimic the selecticity of the nuclear pore complex.. Nature.

[24] Gillespie D, Boda D, He Y, Apel P, Siwy Z (2008). Synthetic nanopores as a test case for ion channel theories: The anomalous mole fraction effect without single filing.. Biophys J.

[25] Berezhkovskii AM, Bezrukov SM (2005). Channel-facilitated membrane transport: Constructive role of particle attraction to the channel pore.. Chem Phys.

[26] Berezhkovskii AM, Bezrukov SM, Pustovoit MA (2002). Channel-facilitated membrane transport: Transit probability and interaction with the channel.. J Chem Phys.

[27] Noble R (1991). Facilitated transport with fixed-site carrier membranes.. J Chem Soc, Faraday Trans.

[28] Noble RD (1992). Generalized microscopic mechanism of facilitated transport in fixed site carrier membranes.. Journal of membrane science.

[29] Cussler EL, Aris R, Bhown A (1989). On the limits of facilitated diffusion.. J Membrane Sci.

[30] Wyman J (1966). Facilitated diffusion and the possible role of myoglobin as a transport mechanism.. J Biol Chem.

[31] Bauer WR, Nadler W (2006). From the Cover: Molecular transport through channels and pores: Effects of in-channel interactions and blocking.. Proc Natl Acad Sci USA.

[32] Cussler E (1997). Diffusion: Mass transfer in fluid systems: Cambridge Univ Pr..

[33] Berezhkovskii A, Bezrukov S (2005). Optimizing Transport of Metabolites through Large Channels: Molecular Sieves with and without Binding.. Biophys J.

[34] Bezrukov SM, Berezhkovskii AM, Szabo A (2007). Diffusion model of solute dynamics in a membrane channel: Mapping onto the two-site model and optimizing the flux.. J Chem Phys.

[35] Bezrukov SM, Berezhkovskii AM, Pustovoit MA, Szabo A (2000). Particle number fluctuations in a membrane channel.. J Chem Phys.

[36] Chou T (1998). How fast do fluids squeeze through microscopic single-file pores?. Phys Rev Lett.

[37] Kolomeisky A (2006). Channel-Facilitated Molecular Transport across Membranes: Attraction, Repulsion, and Asymmetry.. Phys Rev Lett.

[38] Chou T, Lohse D (1999). Entropy-Driven Pumping in Zeolites and Biological Channels.. Phys Rev Lett.

[39] Smit B, Krishna aR (2003). Molecular simulations in zeolitic process design.. Chem Eng Sci.

[40] Bauer W, Nadler W (2005). Stationary flow, first passage times, and macroscopic Fick's first diffusion law: Application to flow enhancement by particle trapping.. The Journal of Chemical Physics.

[41] Karger J (2008). Single-file diffusion in zeolites.. Adsorption and Diffusion..

[42] Zilman A (2009). Effects of Multiple Occupancy and Interparticle Interactions on Selective Transport through Narrow Channels: Theory versus Experiment..

[43] Gardiner M (2003). Stochastic Processes in Physics, Chemistry and Biology: Springer-Verlag..

[44] Derrida B, Domany E, Mukamel D (1992). An exact solution of a one-dimensional asymmetric exclusion model with open boundaries.. J Stat Phys.

[45] Schuetz GM (2003). Critical phenomena and universal dynamics in one-dimensional driven diffusive systems with two species of particles.. J Phys A: Math Gen.

[46] Schuetz GM (2005). Single-file diffusion far from equilibrium.. Diffusion Fundamentals.

[47] Cooper K, Gates P, Eisenberg R (1988). Diffusion theory and discrete rate constants in ion permeation.. Journal of Membrane Biology.

[48] Lakatos G, Chou T (2003). Totally asymmetric exclusion processes with particles of arbitrary size.. J Phys A: Math Gen.

[49] Berezhkovskii AM, Hummer G (2002). Single-File Transport of Water Molecules through a Carbon Nanotube.. Phys Rev Lett.

[50] Berg HC (2001). Random Walks in Biology: Princeton University Press..

[51] Chou T (1999). Kinetics and thermodynamics across single-file pores: Solute permeability and rectified osmosis.. The Journal of Chemical Physics.

[52] Hahn K, Kärger J, Kukla V (1996). Single-file diffusion observation.. Physical review letters.

[53] Schuss Z, Nadler B, Eisenberg RS (2001). Derivation of Poisson and Nernst-Planck equations in a bath and channel from a molecular model.. Phys Rev E.

[54] Bortz A, Kalos M, Lebowitz J (1975). A New Algorithm for Monte Carlo Simulation of Ising Spin Systems.. Journal of Computational Physics.

[55] Gillespie D (1977). Exact stochastic simulation of coupled chemical reactions.. The Journal of Physical Chemistry.

[56] Le Doussal P, Monthus C, Fisher D (1999). Random walkers in one-dimensional random environments: Exact renormalization group analysis.. Physical Review E.

[57] Zilman A, Pearson J, Bel G (2009). Effects of jamming on nonequilibrium transport times in nanochannels.. Physical review letters.

[58] Eijkel J, Berg A (2005). Nanofluidics: what is it and what can we expect from it?. Microfluidics and Nanofluidics.

[59] Peters R (2009). Translocation through the nuclear pore: Kaps pave the way.. BioEssays.

[60] Denning DP, Patel SS, Uversky V, Fink AL, Rexach M (2003). Disorder in the nuclear pore complex: the FG repeat regions of nucleoporins are natively unfolded.. Proceedings of the National Academy of Sciences.

[61] Lim RYH, Fahrenkrog B, Koser J, Schwarz-Herion K, Deng J (2007). Nanomechanical basis of selective gating by the nuclear pore complex.. Science.

[62] Frey S, Görlich D (2007). A saturated FG-repeat hydrogel can reproduce the permeability properties of nuclear pore complexes.. Cell.

[63] Peters R (2005). Translocation Through the Nuclear Pore Complex: Selectivity and Speed by Reduction-of-Dimensionality.. Traffic.

[64] Colwell L, Brenner M, Ribbeck K, Gilson M Charge as a Selection Criterion for Translocation through the Nuclear Pore Complex.. PLoS Comput Biol.

[65] Hopfield J (1974). Kinetic Proofreading: A New Mechanism for Reducing Errors in Biosynthetic Processes Requiring High Specificity.. Proceedings of the National Academy of Sciences.

[66] Ninio J (1975). Kinetic amplification of enzyme discrimination.. Biochimie.

[67] Mckeithan T (1995). Kinetic Proofreading in T-Cell Receptor Signal Transduction.. Proc Natl Acad Sci.

[68] Yang W, Musser SM (2006). Nuclear import time and transport efficiency depend on importin {beta} concentration.. Journal of Cell Biology.

[69] Dange T, Grunwald D, Grunwald A, Peters R, Kubitscheck U (2008). Autonomy and robustness of translocation through the nuclear pore complex: a single-molecule study.. J Cell Biol.

[70] Kubitscheck U, Grünwald D, Hoekstra A, Rohleder D, Kues T (2005). Nuclear transport of single molecules: dwell times at the nuclear pore complex.. The Journal of Cell Biology.

[71] Yang W, Gelles J, Musser S (2004). Imaging of single-molecule translocation through nuclear pore complexes.. Proc Natl Acad Sci USA.

[72] Bel G, Munsky B, Nemenman I The simplicity of completion time distributions for common complex biochemical processes.. Physical Biology.

[73] McClean M, Mody A, Broach J, Ramanathan S (2007). Cross-talk and decision making in MAP kinase pathways.. Nature Genetics.

[74] Zhou H, Wlodek S, McCammon J (1998). Conformation gating as a mechanism for enzyme specificity.. Proc Natl Acad Sci USA.

