# Supplementary material for: Enhancement of Transport Selectivity through Nano-Channels by Non-Specific Competition
Source: PLoS Comput Biol. 2010 Jun 10;6(6):e1000804. doi: 10.1371/journal.pcbi.1000804 (PMC2883555; doi:10.1371/journal.pcbi.1000804)
Supplement: Text S1 — Supporting information text and figure captions. (0.33 MB DOC) [file pcbi.1000804.s001.doc]

**Supporting Information**

**1. Analytical solutions for two site channel**

Understanding the selectivity of transport through nano-channels requires explicitly taking into account the kinetics of transport of the particles inside the channel, from entrance to exit. Let us consider the simplest example, a 'two-site' channel model [1,2,3,4,5]. The kinetics of such a channel is illustrated in Fig. S1. The channel is represented by two sites: 1 and 2. Particles of two different types attempt to enter the channel at site 1, if it is not occupied, with average fluxes $J_{n}$ and $J_{m}$. From position 1, a particle of species $n$ can either hop to position 2, if it is not occupied, with the rate $r_{1\to2}^{n}$, or return left with an average rate $r_{\leftarrow}^{n}$ ($r_{1\to2}^{m}$ and $r_{\leftarrow}^{m}$ for a particle of type $m$). Similarly, from position 2, a particle can either hop left to position 1, if it is not occupied, with a rate $r_{2\to1}^{n}$, or jump forward to the right with an average rate $r_{\to}^{n}$ ($r_{2\to1}^{m}$ and $r_{\to}^{m}$ for particles of species $m$). To simplify the analysis, in this section we shall assume that the channel is uniform: $r_{1\to2}^{n}=r_{2\to1}^{n}=r$ and $r_{1\to2}^{m}=r_{2\to1}^{m}=r$, and symmetric: $r_{\to}^{n}=r_{\leftarrow}^{n}=r_{o}^{n}$ and $r_{\to}^{m}=r_{\leftarrow}^{m}=r_{o}^{m}$.

The channel can be in nine occupancy states (i.e., both sites are free; the first site is occupied by an $n$-species particle and the second is free; the first site is free and the second is occupied by an $n$-species particle, etc.) We denote these states as $00,n0,0n,m0,0m,nm,mn,nn,mm$(Fig. S1). Because of the random nature of hopping between positions in the channel, at any time $t$ the channel is characterized by a probability vector to be in any of the states:

$$\vec{P}(t)=(P_{00}(t),P_{n0}(t),P_{0n}(t),P_{nn}(t),P_{m0}(t),$$

$$P_{0m}(t),P_{mm}(t),P_{nm}(t),P_{mn}(t))$$

Time evolution of these probabilities is described by a Master equation [4,6]

(1.S)

where the kinetic matrix $\hat{M}$ is:

$\hat{M}$=$\left(\begin{matrix} -J_{n}-J_{m} & r_{\leftarrow} & r_{\to} & 0 & q_{\leftarrow} & q_{\to} & 0 & 0 & 0 \\ J_{n} & r-r_{\leftarrow} & r & r_{\to} & 0 & 0 & 0 & q_{\to} & 0 \\ 0 & r & -r-r_{\to}-J_{n}-J_{m} & r_{\leftarrow} & 0 & 0 & 0 & 0 & q_{\leftarrow} \\ 0 & 0 & J_{n} & -r_{\leftarrow}-r_{\to} & 0 & 0 & 0 & 0 & 0 \\ J_{m} & 0 & 0 & 0 & -q_{\leftarrow}-q & q & q_{\to} & 0 & r_{\to} \\ 0 & 0 & 0 & 0 & q & -J_{m}-J_{n}-q-q_{\to} & q_{\leftarrow} & r_{\leftarrow} & 0 \\ 0 & 0 & 0 & 0 & 0 & J_{m} & -q_{\leftarrow}-q_{\to} & 0 & 0 \\ 0 & 0 & 0 & 0 & 0 & J_{n} & 0 & -r_{\leftarrow}-q_{\to} & 0 \\ 0 & 0 & J_{m} & 0 & 0 & 0 & 0 & 0 & -q_{\leftarrow}-r_{\to} \\ & & & & & & & & \end{matrix}\right)$

which corresponds to the kinetic scheme of Fig. 2. In the present notations, $r_{x}=r_{x}^{n}$ and $q_{x}=r_{x}^{m}$. The matrix $\hat{M}$ of (1.S) is degenerate, because

$$\frac{d}{dt}(P_{00}+P_{0n}+P_{n0}+P_{nn}+P_{0m}+P_{m0}+P_{mm}+P_{mn}+P_{nm})=0$$

so that $\det\hat{M}=0$. However, supplemented with a condition $P_{00}+P_{0n}+P_{n0}+P_{nn}+P_{0m}+P_{m0}+P_{mm}+P_{mn}+P_{nm}=1$ that reflects the fact that the channel has to be at *some* state, equations become well defined. At steady state, the solution is then given by the system of linear equations

(2.S)

where $\vec{u}=(1,1,1,1,1,1,1,1,1)$. The transmitted current of the particles of species $n$ is $J_{n}^{\text{out}}=r_{\to}^{n}(P_{0n}+P_{nn}+P_{mn})$, which represents the total flux forward from the states that have a particle of species $n$ at position 2. Similarly, the forward exit current of the $m$-species is $J_{m}^{\text{out}}=r_{\to}^{m}(P_{0m}+P_{mm}+P_{nm})$.

Again, the *efficiency* of transport of the particles of each species is defined as the ratio of the transmitted current to the entering flux: $\text{Eff}_{n}=J_{n}^{\text{out}}/J_{n}$ for $n$-type and $\text{Eff}_{m}=J_{m}^{\text{out}}/J_{m}$ for $m$-species particles.

In the limit of small fluxes, $J\to0$, when only one particle is present at the channel at any time, the transport efficiency becomes identical to the probability that a single particle, starting from position 1, exits to the right [7,8,9,10]. For a non-zero flux, the transport efficiency is dictated by two independent factors: 1) the ability of a particle to enter the channel (since the entrance site is occupied with a probability $1-(P_{00}+P_{0m}+P_{0n})$, only a fraction of the incoming flux $J^{\text{in}}=J(P_{00}+P_{0m}+P_{0n})$ actually enters the channel) and 2) the probability that a single particle translocates through the channel *after* it has entered: $P_{\to}=J^{\text{out}}/J^{\text{in}}$.

**Single species case** First, we review how trapping in the channel influences transport in the case when only one particle species is present (let us say, $n$-type particles, so that $J_{m}=0$). In this case, equations (1.S) can be solved explicitly [6,7,9] and give for the translocation probability

$$P_{\to}=J^{\text{out}}/J^{\text{in}}=\frac{r}{2r+r_{o}}$$

Crucially, the translocation probability$P_{\to}$ is higher for particles with lower exit (‘off’) rate $r_{o}$: transient trapping inside the channel increases the probability of individual particles to translocate through it. This phenomenon is known as facilitated diffusion [11,12,13,14,15,16,17,18,19,20].

However, if the trapping time or the flux is too high, the particles that accumulate inside the channel prevent the entrance of new ones. The total transport efficiency is thus reduced at a low values of the exit rate $r_{o}$ or high values of the entrance flux *J* [7,8,9,10,16], so that

$\text{Eff}^{0}=J^{\text{out}}/J=\frac{rr_{o}}{r_{o}(2r+r_{o})+J(r+r_{o})}$ (3.S)

An important feature of equation (3.S) is that transport efficiency - and therefore transmitted flux - is maximized at a particular value of the exit rate $r_{o}$. This feature, which is much more pronounced for longer channels (i.e., with more sites), provides a mechanism for selectivity; only particles possessing near optimal exit rates are transmitted efficiently, as discussed in the introduction.

**Selectivity enhancement in mixtures of different** **species***.*

Selectivity conditions change when one considers transport in a mixture of two different species of molecules. At steady state, equation (1.S) can be solved analytically to determine the transport efficiency and probability for both types of molecules. The full form of the solution is cumbersome and is not shown. In accord with the results of simulations in the main text the main result of the analytical solution is that in the presence of more strongly trapped species, the transport of more weakly trapped species is inhibited.

The results are presented in Fig. S2. Panel **A** shows that when weakly trapped molecules (here denoted as *m*-type) and strongly trapped molecules (*n*-type) compete for the same positions inside the channel, the transport of *m*-type molecules is significantly inhibited compared to the single-species case. Even more strikingly, the transport of the slower, particles is *enhanced* in the presence of faster, non-specific competition as shown in Fig. S2 **B**.

Why does competition between different particle species for space inside the channel enhance the selectivity of transport? As mentioned above, the total transport efficiency is determined by 1) the probability to enter the channel in the first place, because the position at entrance might be occupied 2) the probability of individual particles to translocate through the channel, after entering. The Fig. S2 **C** shows that it is mostly the translocation probability that is influenced by the competition: translocation probability of faster particles decreases, while the translocation probability of the slower species actually increases due to competition! Blocking of the channel entrance by the particles that are already inside affects the $n$-type and the $m$-type particles equally. The entering fraction of the flux is equal for both species: $J_{m}^{\text{in}}/J_{m}=J_{n}^{\text{in}}/J_{n}=1-(P_{00}+P_{0m}+P_{0n})$. Another striking feature of the competition-driven selectivity enhancement is that the weakly trapped $m$-type particles are excluded from the channel even if their influx $J_{m}$ is much higher than that of the slower particles $J_{n}$, as shown in Fig. S2.

**2. Additional information for Fig. 4 in the main text.**

Here we show a different rendering of the results presented in Fig. 4 in the main text, which shows the actual numerical values of the parameters and un-normalized efficiencies and probabilities – Fig S3.

**3. Sensitivity to the choice of the kinetic parameters of the channel**

The effect described in the paper has been observed for various choices of the kinetic profile. Combined with the analyitical derivations (see above), we expect the effect to play the role in the transport selectivity of any transport channel, significantly narrow in order that the particles of different species have to compete for the space inside the channel. Supplementrary data are shown in Fig. S4, for several other kinetic profiles (that may correspond to different physical situations near the channel entrance) and different values of the exit rate of the strongly trapped sepcies; All of them exhibit the enhancement of the selectivity by non-specific competition.

**4. Simulation algorithm**

We use a variant of the Gillespie (Kinetic Monte Carlo) algorithm[21,22,23]. At each step of the simulation, an $n$-type particle that is at a position *i* inside the channel, hops to an adjacent position $i\pm1$ with the probability

$$P_{i\to i\pm1}^{n}=\frac{R_{i\to i\pm1}}{J_{n}+J_{m}+\sum_{i} \left[R_{i\to i\pm1}+Q_{i\to i\pm1}\right]}$$

where we have denoted the actual transition rates, which take into account the site occupancies, as $R_{i\to i\pm1}=n_{i}r_{i\to i\pm1}(1-o(i\pm1))$ and $Q_{i\to i\pm1}=m_{i}q_{i\to i\pm1}(1-o(i\pm1))$; the number of $n$-type particles at site $i$ is $n_{i}$ and the number of $m$-type particles is $m_{i}$. The occupancy of a position $i$ is $o(i)=0$ if the position $i$ is not fully occupied, and $o(i)=1$ if the position is occupied by a maximal allowed number of particles of any type. The hopping of the $m$-type particles is described with an analogous expression (see the actual code). The exit and entrance reactions are treated in a similar way. See the code for details (attached).

The simulation algorithm was validated against known analytical solutions in two cases: i) for two-site channel with competition (solutions of equation (1.S) shown in Fig. S1) and ii) for a particular case of single-species transport through a long channel [41]. Perfect agreement between the simulation and the analytical solutions has been found (data not shown).

**5. Supplementary data for Fig. 7**

Although the presence the weakly trapped species enhances the transport efficiency of the more strongly trapped one even when the former is present in high excess, eventually for very high concentrations of the weakly trapped species, it clogs the channel and the transport of the stronly trapped one diminishes. Thus, for given trapping strengths of both species, the transport efficiency of the strongly trapped one exhibits a maximum upon progressive addition of the non-specific one. On the other hand, in the case of titration, the selectivity enhancement increases with the total particle concentration – as might be expected, because at high concentration the competition for space is more pronounced. See Fig. S5. A comprehensive study of different regimes is beyond of the scope of this work and will be presented elsewhere.

**Simulation code**

/* Two-species exclusion process*/

#include<stdio.h>

#include<math.h>

#include<time.h>

#define LENGTH 10000000

#define capacity 1

/*random number generator */

static int A[4];

static int B[4]={6698,7535,26792,30140};

/* 246913578*(2^32+1)=1060485742695258666 */

#define M0 17917 /* 13^13=302875106592253 */

#define M1 13895

#define M2 19930

#define M3 8

#define PW2 32767 /* 2^(15)-1 */

#define rpw2a 9.31322574615478516e-10 /* 2^(-30) */

#define rpw2b 1.11022302462515654e-16 /* 2^(-53) */

#define rpw2c 2.77555756156289135e-17 /* 2^(-55) */

double Xrandom(void)

{

A[0]=B[0]*M0;

A[1]=(A[0]>>15)+B[1]*M0+B[0]*M1;

A[2]=(A[1]>>15)+B[2]*M0+B[1]*M1+B[0]*M2;

A[3]=(A[2]>>15)+B[3]*M0+B[2]*M1+B[1]*M2+B[0]*M3;

B[0]=A[0]&PW2;

B[1]=A[1]&PW2;

B[2]=A[2]&PW2;

B[3]=A[3]&PW2;

return rpw2a*((B[3]<<15)+B[2])+rpw2b*((B[1]<<8)+(B[0]>>7))+rpw2c;

}

void init_random(int seed)

{

if (seed==0) seed=(int)time(NULL);

B[0]=(2+(seed<<2))&PW2;

B[1]=(seed>>13)&PW2;

B[2]=(seed>>28)&PW2;

B[3]=0;

Xrandom();

}

/*double occup(int x, int y) {

if (x+y < capacity) return 1;

if (x+y >= capacity) return 0;

} */

double occup(int x, int y, int cap) {

return (double)(1-(double)(x+y)/cap);

}

int main(){

int k,i,j,l,L=10,nn,xx,yy,x0=1;

double rate, ratey,t,flip,flipent,cumultot,Nf,Nb,Nr,Nin,Niny,Nfy,Nby,Nry,J=0.01,Jy=0.01,b,f,by,fy;

char nome1[100],nome2[100];

FILE *file1;

int x[L+1],y[L+1];

double pf[L+1],pb[L+1],pfy[L+1],pby[L+1],grates[4*L+4],cumul[4*L+4];

init_random(0);

rate=0;

ratey=0;

file1=fopen("output.txt","w");

fprintf(file1,"%lf\t %lf\t %lf\t \n",J,Jy,ratey);

for(l=0;l<=100;l++){/* loop over different rates*/

rate=0.01*l;

b=1;

f=1;

ratey=0.1;

by=1;

fy=1;

printf("%lf\t %lf\t %lf\t \n",J,Jy,ratey);

/*jumping probabilities definitions*/

for(j=1;j<=L;j++){

pf[j]=1;

pfy[j]=1;

pb[j]=1;

pby[j]=1;

pf[j]=1;

pfy[j]=1;

pb[j]=1;

pby[j]=1;

}

pfy[L]=ratey;

pfy[L-1]=by;

pby[L]=fy;

pby[1]=ratey;

pfy[1]=fy;

pby[2]=by;

pf[L]=rate;

pf[L-1]=b;

pb[L]=f;

pb[1]=rate;

pf[1]=f;

pb[2]=b;

x[0]=0;

y[0]=0;

for(j=1;j<=L;j++){

x[j]=0;

y[j]=0;

}

for(i=0;i<=4*L+3;i++){

grates[i]=0;

cumul[i]=0;

}

t=0;

Nf=0;

Nb=0;

Nr=0;

Nin=0;

Niny=0;

Nfy=0;

Nby=0;

Nry=0;

pr1=0;

while(t<LENGTH){/*start loop over time*/

grates[0]=Jy*occup(x[x0],y[x0],capacity); /*define the bare gillespie rates */

grates[4*L+1]=Jy*(1-occup(x[x0],y[x0],capacity));

grates[4*L+2]=J*(1-occup(x[x0],y[x0],capacity));

grates[4*L+3]=J*occup(x[x0],y[x0],capacity);

for(i=1;i<L;i++){

grates[i]=(double) pf[i]*x[i]*occup(x[i+1],y[i+1],capacity);

grates[2*L+i]=(double) pfy[i]*y[i]*occup(x[i+1],y[i+1],capacity);

}

for(i=2;i<=L;i++){

grates[i+L-2]=(double) pb[i]*x[i]*occup(x[i-1],y[i-1],capacity);

grates[i+3*L-2]=(double) pby[i]*y[i]*occup(x[i-1],y[i-1],capacity);

}

grates[2*L-1]=(double)pf[L]*x[L];

grates[4*L-1]=(double)pfy[L]*y[L];

grates[2*L]=(double)pb[1]*x[1];

grates[4*L]=(double)pby[1]*y[1];

for(i=0;i<=4*L+3;i++){ /*define the cumulative rates */

cumul[i]=0;

}

for(i=0;i<=4*L+3;i++){

for(j=0;j<=i;j++){

cumul[i]+=grates[j];

}

}

cumultot=cumul[4*L+3];

for(i=0;i<=4*L+3;i++){

cumul[i]= cumul[i]/cumultot;

}

flip=Xrandom();/*start gillespie simulation*/

if(flip<cumul[0]){

y[x0]=1;

Niny+=1;

}

else if(flip>cumul[4*L+2]){

x[x0]=1;

Nin+=1;

}

else if((flip>cumul[4*L])&&(flip<cumul[4*L+1])){

Nry+=1;

}

else if((flip>cumul[4*L+1])&&(flip<cumul[4*L+2])){

Nr+=1;

}

else{

for(i=1;i<=4*L;i++){

if((flip>cumul[i-1])&&(flip<cumul[i]))

{

if(i<L){

x[i+1]+=1;

x[i]=x[i]-1;

}

else if((i>L-1)&&(i<=2*L-2)){

x[i-L+1]+=1;

x[i-L+2]=x[i-L+2]-1;

}

else if(i==2*L-1){

Nf+=1;

x[L]=x[L]-1;

}

else if (i==2*L){

Nb+=1;

x[1]=x[1]-1;

}

else if ((i>2*L)&&(i<3*L)){

y[i-2*L+1]+=1;

y[i-2*L]=y[i-2*L]-1;

}

else if ((i>3*L-1)&&(i<=4*L-2)){

y[i-3*L+1]+=1;

y[i-3*L+2]=y[i-3*L+2]-1;

}

else if (i==4*L-1){

Nfy+=1;

y[L]=y[L]-1;

}

else {

Nby+=1;

y[1]=y[1]-1;

}

}/*closes inner i if*/

}/*closes loop over i*/

}/* end gillespie updating */

t+=1/(cumultot)*log(1/Xrandom());/*update gillespie time */

}/*closes loop over t*/

fprintf(file1,"%lf %lf %lf %lf %lf %lf %lf\n",rate,ratey,Nf/(Nb+Nf),Nf/(Nf+Nb+Nr),Nfy/(Nby+Nfy),Nfy/(Nfy+Nby+Nry));/*print output into file*/

printf("%lf %lf %lf %lf %lf %lf %lf\n",rate,ratey,Nf/(Nb+Nf),Nf/(Nf+Nb+Nr),Nfy/(Nby+Nfy),Nfy/(Nfy+Nby+Nry));

}/*closes loop over rates*/

} /*closes main*/

**References**

1. Bezrukov SM, Berezhkovskii AM, Szabo A (2007) Diffusion model of solute dynamics in a membrane channel: Mapping onto the two-site model and optimizing the flux. J Chem Phys 127: 115101.

2. Schuetz GM (2003) Critical phenomena and universal dynamics in one-dimensional driven diffusive systems with two species of particles. J Phys A: Math Gen 36: R339-R379.

3. Chou T, Lohse D (1999) Entropy-Driven Pumping in Zeolites and Biological Channels. Phys Rev Lett 82: 3552-3555.

4. Chou T (1999) Kinetics and thermodynamics across single-file pores: Solute permeability and rectified osmosis. The Journal of Chemical Physics 110: 606.

5. Kolomeisky A (2006) Channel-Facilitated Molecular Transport across Membranes: Attraction, Repulsion, and Asymmetry. Phys Rev Lett 98: 048105.

6. Gardiner M (2003) Stochastic Processes in Physics, Chemistry and Biology: Springer-Verlag.

7. Chou T (1998) How fast do fluids squeeze through microscopic single-file pores? Phys Rev Lett 80: 85-88.

8. Berezhkovskii A, Bezrukov S (2005) Optimizing Transport of Metabolites through Large Channels: Molecular Sieves with and without Binding. Biophys J 88: L17-L19.

9. Zilman A (2008) Effects of multiple occupancy and inter-particle interactions on selective transport through narrow channels: theory vs. experiment. Biophys J in press.

10. Zilman A, Di Talia S, Chait B, Rout M, Magnasco M, et al. (2007) Efficiency, Selectivity, and Robustness of Nucleocytoplasmic Transport. PLoS Comput Biol 3: e125.

11. Noble R (1991) Facilitated transport with fixed-site carrier membranes. J Chem Soc, Faraday Trans 87: 2089-2092.

12. Noble RD (1992) Generalized microscopic mechanism of facilitated transport in fixed site carrier membranes. Journal of membrane science 75: 121-129.

13. Berezhkovskii AM, Bezrukov SM (2005) Channel-facilitated membrane transport: Constructive role of particle attraction to the channel pore. Chem Phys 319: 342.

14. Berezhkovskii AM, Bezrukov SM, Pustovoit MA (2002) Channel-facilitated membrane transport: Transit probability and interaction with the channel. J Chem Phys 116: 9952-9956.

15. Wyman J (1966) Facilitated diffusion and the possible role of myoglobin as a transport mechanism. J Biol Chem 211: 114-121.

16. Bauer WR, Nadler W (2006) From the Cover: Molecular transport through channels and pores: Effects of in-channel interactions and blocking. Proc Natl Acad Sci USA 103: 11446-11451.

17. Cussler EL, Aris R, Bhown A (1989) On the limits of facilitated diffusion. J Membrane Sci 43: 149-164.

18. Lee SB, al. e (2002) Antibody-based bio-nanotube membranes for enantiomeric drug separations. Science 296: 2198-2200.

19. P. Kohli ea (2004) DNA-Functionalized Nanotube Membranes with Single-Base Mismatch Selectivity. Science 305: 984-986.

20. Jirage KB, Hulteen JC, Martin CR (1997) Nanotubule-Based Molecular-Filtration Membranes. Science 278: 655.

21. Gillespie D (1977) Exact stochastic simulation of coupled chemical reactions. The Journal of Physical Chemistry 81: 2340-2361.

22. Bortz A, Kalos M, Lebowitz J (1975) A New Algorithm for Monte Carlo Simulation of Ising Spin Systems. Journal of Computational Physics 17: 10.

23. Le Doussal P, Monthus C, Fisher D (1999) Random walkers in one-dimensional random environments: Exact renormalization group analysis. Physical Review E 59: 4795-4840.

**Figure captions**

Figure S1 **Kinetic diagram of transport of particles of two different species through a two-site channel.** **A.** Kinetic diagram of a channel consisting of two positions. **B.** Occupancy representation: transition scheme between nine occupancy states: $00,n0,0n,m0,0m,nm,mn,nn,mm$.

Figure S2 **Selectivity enhancement in a mixture of two species: two site channel**

**A.** Transport of strongly trapped particles is *enhanced* by the competition with the faster ones. The dotted line shows the transport efficiency of the strongly trapped species of exit rate $r_{o}^{n}/r=0.1$ without competition. The blue line shows transport efficiency of the slower particles, in competition with faster particles, as a function of the exit rate of the faster particles $r_{o}^{m}/r$, present in the same amount, ($J_{m}=J_{n}$). The enhancement occurs even to a higher degree in the 9:1 excess of the fast particles ($J_{m}=0.9J, J_{n}=0.1J$) - turquoise line. **B.** Transport of the weakly trapped species is inhibited by the competition with the stringly trapped one. The dotted line shows the transport efficiency of the weakly trapped species as a function of their exit rate without competition. The black line shows transport efficiency of the weakly trapped particles as a function of their exit rate $r_{o}^{m}/r$, in competition with strongly trapped particles whose exit rate is kept fixed at $r_{o}^{n}/r=0.1$, present in the same amount, ($J_{m}=J_{n}$). Dotted line- no competition. The inhibition occurs even in the 9:1 excess of the fast particles ($J_{m}=0.9J, J_{n}=0.1J$) - gray line. **C.** The probability of a particle of weakly trapped species to translocate through the channel is diminished in the presence of the slower particles. By contrast, the probability of a particle of strioingly trapped species to translocate through the channel is enhanced in the mixture. Dotted line - no competition, black line- 1:1 mixture ($J_{m}=J_{n}$), gray line: 9-fold excess of the faster particles ($J_{m}=0.9J, J_{n}=0.1J$). **D.** Ratio of the transport efficiency of the weakly trapped species to that of the strongly trapped species. Dotted line- no competition. Black line:equal mixture ($J_{m}=J_{n}$), gray line: 9-fold excess of the weakly trapped particles ($J_{m}=0.9J, J_{n}=0.1J$). In all panels $J=0.1$.

Figure S3 **Selectivity enhancement in a mixture of two species: long channel** In all panels the exit rate of the strongly trapped species is kept fixed $r_{o}^{n}/r=0.1$ and the total flux $J/r=J_{m}/r+ J_{n}/r=0.01$**A.** The blue line shows transport efficiency $\text{Eff}_{\text{m}}$of the weakly trapped particles as a function of their exit rate $r_{o}^{m}/r$, in competition with strongly trapped particles present in the same amount, ($J_{m}=J_{n}$). Dotted line- no competition. Turquoise line – 9:1 excess of the weakly trapped species $J_{m}=0.9J, J_{n}=0.1J$. **B.** Probability of translocation through the channel of the weakly trapped species $\text{P}_{\to}^{\text{m}}$. **C.** Probability to enter the channel of the weakly trapped species, $\text{P}_{in}^{\text{m}}$. Dotted line- no competition. Turquoise line – 9:1 excess of the weakly trapped species $J_{m}=0.9J, J_{n}=0.1J$. **D.** Efficiency of transport of the strongly trapped species in competition with more weakly trapped one, $\text{Eff}_{\text{n}}$. **E.** Probability of translocation through the channel of the strongly trapped species, $\text{P}_{\to}^{\text{n}}$ **F.** Probability of the strongly trapped species to enter the channel, $\text{P}_{in}^{\text{n}}$. In all panels **D, E, F,** the black line represents 1:1 mixture ($J_{m}=J_{n}$) and the gray line represents 9:1 excess of the weakly trapped particles ($J_{m}=0.9J, J_{n}=0.1J$). Note that the absolute value of the entrance probability is identical for both species (panels **C** and **F)**.

Figure S4 **Sensitivity to the choice of the kinetic profile**

Ratios of the transport efficiency of the weakly trapped species to the transport efficiency of the strongly trapped species for the different kinetic profiles shown in the insets to each panel for different values of the exit rate of the strongly trapped species. See text in Section 3 of the Supplementary Material for discussion.

Figure S5 **Dependence of the selectivity on the concentration**

Panel **A:** Transport efficiency of the strongly trapped species as a function of the flux of the weakly trapped species, in the case of *addition*. $J_{n}/r=0.01$ , $r_{o}^{n}/r=0.1$, $r_{o}^{m}/r=0.3$ Panel **B**: Ratio of transport efficiencies of the weakly trapped species and the strongly trapped species, relative to the no-competition case, as a function of the total flux of the particles of both species, for the case of *titration*. The ratio of the concentrations is 1:1, $r_{o}^{n}/r=0.1$, $r_{o}^{m}/r=0.3$,$J/r=J_{m}/r+ J_{n}/r=0.01$
